# Observations on spiny dogfish ( *Squalus acanthias*) captured in late spring in a North Carolina estuary

**DOI:** 10.12688/f1000research.4890.2

**Published:** 2014-10-13

**Authors:** Charles Bangley, Roger Rulifson

**Affiliations:** 1 Institute for Coastal Science and Policy, East Carolina University, Flanagan 250, East 5th St., Greenville, NC, 27858, USA

## Abstract

Five spiny dogfish were captured in early-mid May during gillnet and longline sampling targeting juvenile coastal sharks in inshore North Carolina waters.  Dogfish captures were made within Back Sound and Core Sound, North Carolina. All dogfish were females measuring 849-905 mm total length, well over the size at 50% maturity. Dogfish were caught at stations 1.8-2.7 m in depth, with temperatures 22.9-24.2 °C, 32.8-33.4 ppt salinity, and 6.9-8.0 mg/L dissolved oxygen. These observations are among the latest in the spring for spiny dogfish in the southeastern U.S. and occurred at higher temperatures than previously recorded for this species.  It is unclear whether late-occurring spiny dogfish in this area represent a cryptic late-migrating or resident segment of the Northwest Atlantic population.

## Introduction

The spiny dogfish ( *Squalus acanthias*) is a small, highly migratory coastal shark common in Northwest Atlantic waters from Newfoundland to Cape Hatteras ( Stehlik, 2007). After signs of population disturbance resulting from overfishing, stringent fishery management regulations were put in place for this species, and the population was considered recovered within ten years of implementing a fishery management plan ( Rago & Sosebee, 2010). Such a swift recovery was unexpected for this species due to its life history characteristics: spiny dogfish in the Northwest Atlantic are not reproductively mature until an age of 12 years, have a 2-year gestation period, and give birth to only 1–15 young ( Nammack *et al.*, 1985).

A possible hypothesis for swifter than expected recovery is that currently cryptic migratory behavior moves some portions of the spiny dogfish population out of range of both fishing pressure and fishery-independent surveys used to assess spiny dogfish stocks. According to data from the National Marine Fisheries Service (NMFS) trawl survey, spiny dogfish exhibit a general north-south migration pattern along the U.S. Atlantic coast, occurring in North Carolina and Virginia waters south to Cape Hatteras during the winter and spring, and moving north to the Gulf of Maine and Canadian waters in the summer and fall ( Stehlik, 2007). However, spiny dogfish movements and distribution may not conform to this pattern. Mark-recapture studies based in both the U.S. and Canada provide evidence for more complex migratory behavior, with little migratory overlap between the Gulf of Maine and Atlantic waters south of Cape Cod and inshore-offshore migrations among dogfish remaining in Canadian waters year-round ( Campana *et al.*, 2007). More recently, spiny dogfish tagged with pop-up satellite tags in the Gulf of Maine were tracked moving off the continental shelf, providing more evidence for inshore-offshore migrations ( Sulikowski *et al.*, 2010).

Spiny dogfish also occur south of Cape Hatteras, with large aggregations encountered during the winter and early spring from Cape Lookout to Cape Fear in North Carolina waters ( Rulifson & Moore, 2009) and along the South Carolina coast ( Ulrich *et al.*, 2007). Spiny dogfish south of Cape Hatteras tend to occur in shallower water closer to shore than conspecifics north of Cape Hatteras ( Rulifson & Moore, 2009; Rulifson *et al.*, 2012). Acoustic telemetry data suggest that these sharks are part of the population that migrates between Cape Hatteras and Cape Cod ( Rulifson *et al.*, 2012), and seem to occupy southern waters between November and April ( Ulrich *et al.*, 2007; Rulifson *et al.*, 2012). Despite this consistent behavior among acoustically tagged sharks, Rulifson *et al.*, (2012) reported the capture of several spiny dogfish by hook and line at Cape Lookout on June 1, 2010, long after the end of the overwintering period for this species, though environmental measurements were not recorded. Here we report further observations of spiny dogfish occurring in southern waters long after their expected migration north.

## Methods

**Figure 1.**
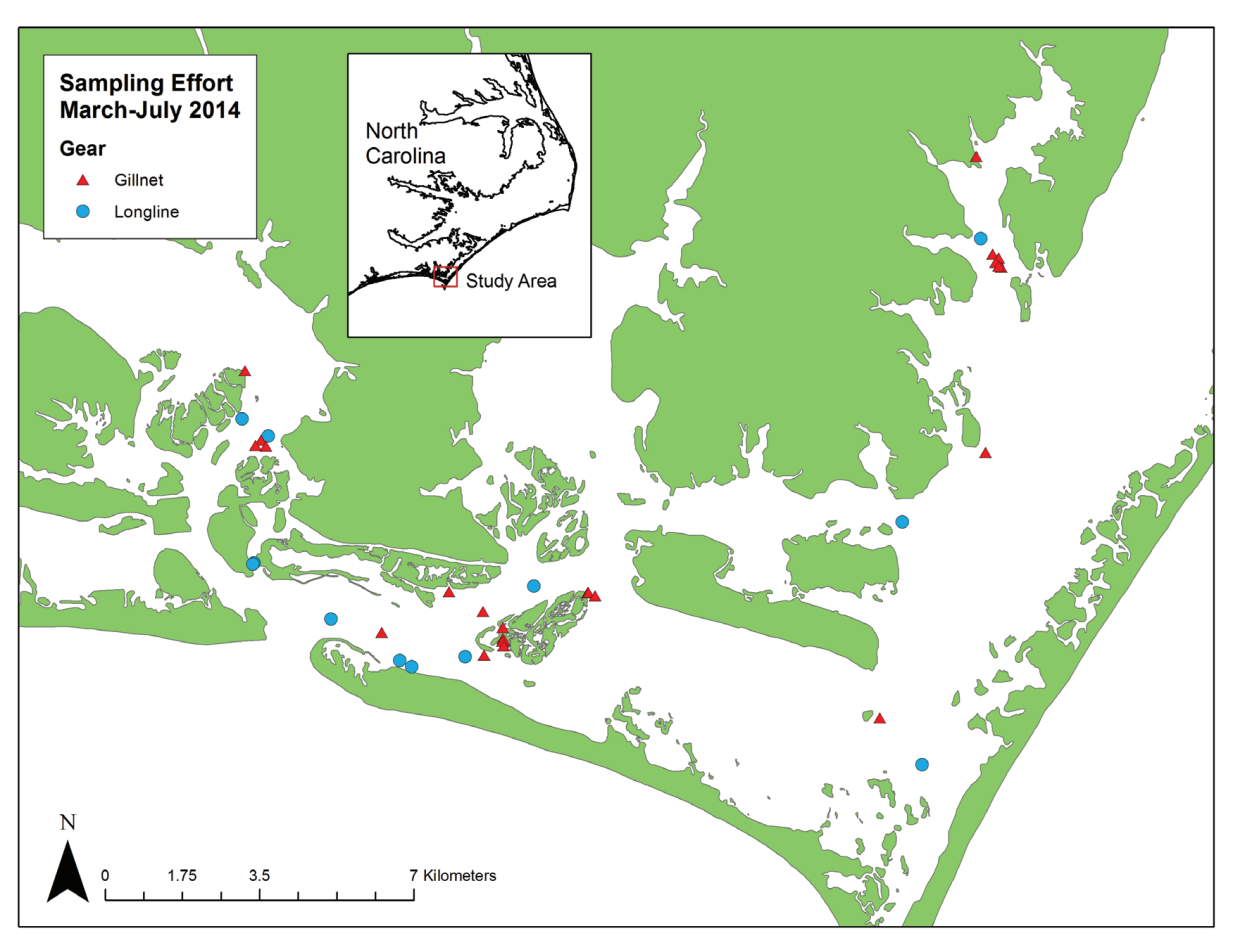
Gillnet and longline sampling stations within Back Sound and lower Core Sound from March 21 – July 1, 2014.

Spiny dogfish were captured during a survey designed to assess habitat selection by juvenile coastal sharks in North Carolina inshore waters. The sampling area encompassed the entirety of Back Sound from Beaufort Inlet to Cape Lookout, and extended north through the southern extent of Core Sound into Jarrett Bay ( Figure 1). Sampling also occurred within Newport River from Beaufort Inlet to the Newport Marshes.

Sharks were captured using bottom-set gillnet gear. Gillnet gear measured 50 m in length and 2.4 m in height, and was comprised of eight panel sections of monofilament mesh measuring 7.5, 10, 12.3, 15.5, 17.1, 21, 25.6, and 31 cm stretched, respectively, and was soaked for 30–60 minutes. At each sampling location, depth (m) was recorded using an onboard depth sounder, and temperature (°C), salinity (ppt), and dissolved oxygen (mg/L) were measured using a YSI model 85.

All captured sharks were identified to species and sex, fork length (FL, mm), and stretched total length (TL, mm) were recorded. Signs of life-history stage such as umbilical scarring and visible pregnancy were also recorded.

## Results

A total of 52 stations were sampled from March 21 to July 1, 2014, 12 of which were sampled using longline gear and 31 of which were sampled by gillnet. Sampling encompassed Newport River, the western half of Back Sound through Middle Marsh, and Core Sound between Cape Lookout and Jarrett Bay ( Figure 1).

**Figure 2.**
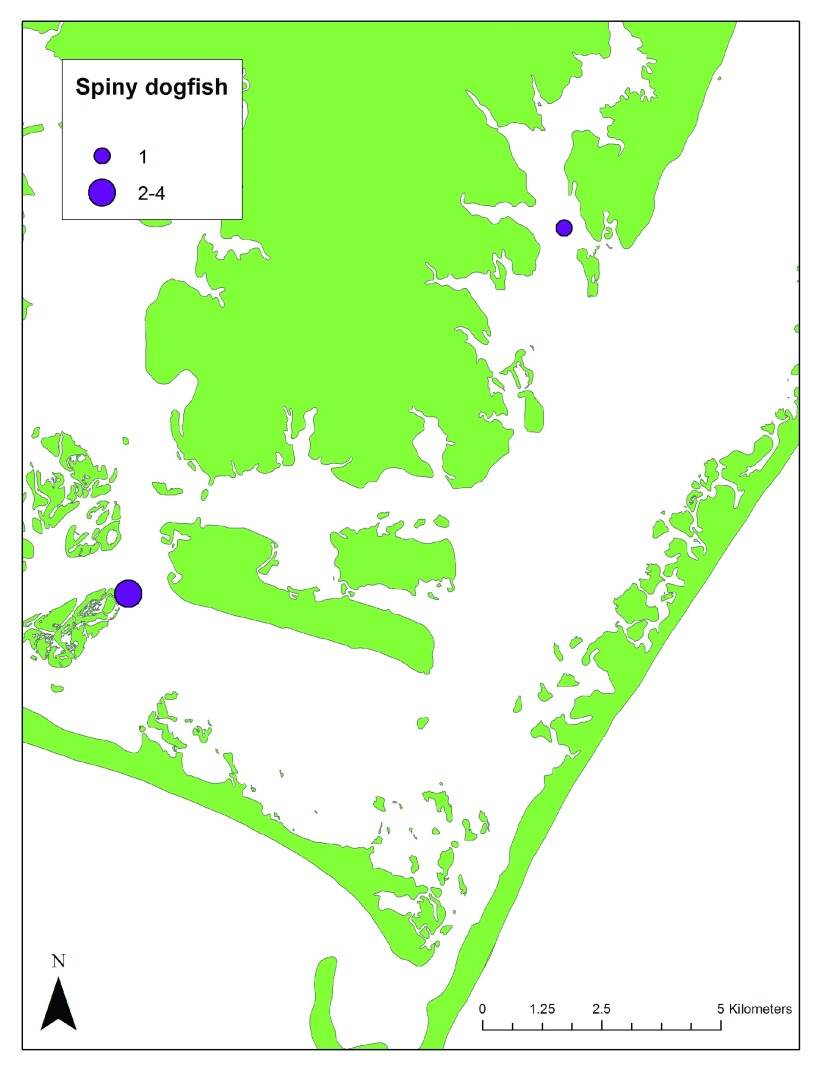
Capture locations of spiny dogfish in gillnet sets within Back Sound and lower Core Sound in May 2014.

Spiny dogfish were captured in May during two gillnet sets ( Figure 2, Data Set 1). The first capture event took place on May 6 during a gillnet set deployed at the northeast corner of Middle Marsh. The gear was deployed at 1325 hours and allowed to soak for 30 minutes. Four adult female spiny dogfish ranging from 849–905 mm TL were captured. The site of capture was 2.74 m in depth, with a temperature of 22.9°C, salinity of 32.8 ppt, and 8.0 mg/L dissolved oxygen.

Date, time, and location of spiny dogfish captures in gillnet gear, with size, sex, and environmental data taken at each station TL: total length; FL: fork length. The last spiny dogfish escaped alongside boat.

The second capture event occurred on May 18 on the north side of Davis Island in Jarrett Bay. Time of gillnet deployment was 1442 hours and soak time was limited to 30 minutes. One spiny dogfish was snared in the mesh by its dorsal spines but was able to break free and escape before it could be brought aboard. Visual estimate placed the TL of this shark within the range of those captured earlier (850–900 mm). A depth of 1.8 m, temperature of 24.2°C, salinity of 33.4 ppt, and dissolved oxygen of 6.9 mg/L were recorded at this site.

## Discussion

These observations represent the highest reported temperatures and latest occurrence for spiny dogfish in their overwintering habitat off the Southeastern U.S, with the exception of those captured on June 1, 2010 by Rulifson *et al.*, (2012). The presence of these sharks in North Carolina waters in late May at temperatures above 22°C is inconsistent with current information on spiny dogfish distribution and environmental preferences. Whether these observations point to unique behavior among spiny dogfish occurring near Cape Lookout or the limitations of other sampling efforts for this species is uncertain.

Spiny dogfish have been consistently observed overwintering south of Cape Lookout. Bearden, (1965) reported that spiny dogfish were captured in trawl surveys within South Carolina waters as far south as Port Royal Sound between December and March at water temperatures ranging 7.5–12.0°C. Year-round gillnet and longline sampling along the South Carolina coast only captured spiny dogfish at temperatures below 14°C between January and March ( Ulrich *et al.*, 2007). In the vicinity of Cape Fear, North Carolina, Thorpe & Beresoff, (2000) captured spiny dogfish in commercial gillnet gear from December–April, though the sharks were most abundant in February and March and at temperatures less than 13.9°C. In contrast, only one spiny dogfish was captured during gillnet sampling from May-September in the same area ( Thorpe *et al.*, 2004). Schwartz, (2003) reported that spiny dogfish could occasionally be encountered along the coast of the Carolinas until May, but temperatures higher than 18°C triggered migration offshore and northward.

Seasonal habitat preferences inferred from trawl survey and mark/recapture studies focused on the area between Cape Hatteras and the Scotian Shelf are consistent with observations from areas further south. Spiny dogfish occurring between Cape Hatteras and the Gulf of Maine mostly occurred in North Carolina waters during winter and spring, and were distributed between New England and Canadian waters during summer and autumn ( Campana *et al.*, 2007; Stehlik, 2007). Within this area, spiny dogfish were captured primarily in the 5–17°C temperature range ( Sagarese *et al.*, 2014). Spiny dogfish occurred at temperatures up to 20°C in Massachusetts inshore waters in autumn, but were most abundant within the 6–15°C range ( Stehlik, 2007). Acoustically-tagged spiny dogfish were only detected near the Hatteras Bight between mid-December and early April, with the majority of detections occurring in February and March, and appeared to make inshore-offshore movements in search of cooler temperatures ( Rulifson *et al.*, 2012).

Though the observed presence of spiny dogfish was inconsistent with previously documented environmental preferences, observations were consistent with other aspects of spiny dogfish behavior. All of the measured sharks were female and well within size at maturity for this species (799 mm TL, Nammack *et al.*, 1985). This is consistent with observations from nearshore South Carolina waters, where 91.9% of spiny dogfish captured during shark surveys were females, and 80% of females were mature ( Ulrich *et al.*, 2007). Size is inversely correlated with depth in spiny dogfish, with the largest individuals occurring in shallow, nearshore waters ( Methratta & Link, 2007), and mature females occur at significantly higher temperatures and lower depths than other demographic groups ( Sagarese *et al.*, 2014). Dell’Apa *et al.*, (2014) observed a greater proportion of females among spiny dogfish captured by gillnet and longline in Massachusetts Bay, an area with a gradually sloping depth profile, than along the eastern shore of Cape Cod, where depth drops rapidly near shore.

It is unclear whether these late-occurring spiny dogfish represent a fluke occurrence or previously unrecognized behavior. Spiny dogfish remaining within North Carolina waters into May and June have also been reported by Rulifson *et al.*, (2012) and Schwartz, (2003), but have not been documented by most studies. The NMFS seasonal trawl surveys only sample North Carolina waters during the early spring and autumn and may not account for spiny dogfish occurring in the area at other times of the year, but migration out of southern waters in spring has also been suggested by gillnet and longline surveys capable of capturing sharks year-round ( Thorpe & Beresoff, 2000; Thorpe *et al.*, 2004; Ulrich *et al.*, 2007), as well as acoustic telemetry ( Rulifson *et al.*, 2012). Our observations also represent the highest temperatures reported for this species in the southeastern U.S., suggesting that the thermal range for this species may be wider than previously estimated.

Previous studies have shown that spiny dogfish migration and habitat use patterns may be more complex than previously thought ( Campana *et al.*, 2007; Rulifson & Moore, 2009; Sulikowski *et al.*, 2010). The presence of a late-migrating or resident population segment near Cape Lookout may have important implications for spiny dogfish fishery management. Future year-round surveys conducted in tandem with telemetry studies focused on late-season individuals may help explain the unusual spiny dogfish behavior in this area.

## Data availability

F1000Research: Dataset 1. Date, time, and location of spiny dogfish captures in gillnet gear, with size, sex, and environmental data taken at each station, 10.5256/f1000research.4890.d33066 ( Bangley & Rulifson, 2014).
