# Supplementary material for: Observations on spiny dogfish ( Squalus acanthias) captured in late spring in a North Carolina estuary
Source: F1000Res. 2014 Oct 13;3:189. Originally published 2014 Aug 12. [Version 2] doi: 10.12688/f1000research.4890.2 (PMC4240240; doi:10.12688/f1000research.4890.2)
Supplement: Peer review history

# Peer review: Referee response for version 2

**DOI:** 10.5256/f1000research.5671.r6426

**Published:** 2014-11-06

**Authors:** Walter Bubley

**Affiliations:** 1 Marine Resources Research Institute, South Carolina Department of Natural Resources, Charleston, USA

The authors' revisions improve the strength and focus of this manuscript. I only have suggestions for minor changes within the text.

- While mention of the longline component of the survey was removed in the methods section, there is still a reference to it in the first sentence of the results section and in figure 1. My suggestion would be to remove the reference in that first sentence as well as remove Figure 1 altogether. The key part of your paper is that these were caught in higher temperature waters; how the survey was standardized and the location of all of the non-catches is not relevant. I feel like all of the information in the two map figures could be condensed to one if the authors would prefer to keep the non-catch locations in, but I would still recommend removing the longline locations. 

I have read this submission. I believe that I have an appropriate level of expertise to confirm that it is of an acceptable scientific standard.

# Peer review: Referee response for version 1

**DOI:** 10.5256/f1000research.5222.r6101

**Published:** 2014-09-22

**Authors:** Walter Bubley

**Affiliations:** 1 Marine Resources Research Institute, South Carolina Department of Natural Resources, Charleston, USA

This is an informative manuscript detailing previously unrecorded maximum water temperatures in which Spiny Dogfish, *Squalus acanthias*, have been encountered during a standardized survey off the coast of North Carolina. I believe though that the authors need to pare back the content in the methods section to that relevant to the main focus of the paper and not overstate the findings as they relate to habitat use within the estuary in the results and discussion sections. The information obtained in this manuscript has importance in documenting previously unknown temperatures but the authors need to understand that this is the importance and not try to interpret minimal data for habitat use.

- Abstract
  - Remove sentence beginning with "Stations where dogfish..." as there are not enough samples to make any sort of inference to estuary or habitat use
- Methods
  - Remove the sentence in the first paragraph beginning with "Sampling locations were chosen..." as this is not relevant to the capture of the Spiny Dogfish.
  - Remove the mention and description of longline gear use in the second paragraph, as the spiny dogfish were all caught using gillnets. While the survey may have utilized both gear types, the Spiny Dogfish focused on in this paper were not caught with longline.
  - Remove all sentences beginning with "Distance from the nearest inlet..." through the remainder of the second paragraph, as this information is not utilized in the analysis.
  - Remove the sentences beginning with "All batoids were identified..." through the end of the paragraph as this is not relevant to the topic of this manuscript.
- Results
  - Remove all mention of other species captured concurrently with Spiny Dogfish.
  - Remove sentences referring to capture site in relation to inlets and seagrass areas. There is not enough information to do analysis in regards to habitat use.
- Discussion
  - Remove the sentence in the fourth paragraph beginning with "Spiny dogfish feed primarily on..." as this is way too speculative based on the catch of a few harvest fish in one net that also caught spiny dogfish.
  - Remove the fourth paragraph as there are too few samples collected to make any inferences about habitat use for this manuscript.

In general, the manuscript provides useful information in regards to new maximum temperatures recorded for spiny dogfish catch, but I want to emphasize that I would caution the authors from making too many speculative assertions based on limited catch at these temperatures.

I have read this submission. I believe that I have an appropriate level of expertise to confirm that it is of an acceptable scientific standard, however I have significant reservations, as outlined above.

# Peer review: Referee response for version 1

**DOI:** 10.5256/f1000research.5222.r5786

**Published:** 2014-09-10

**Authors:** Bryan Frazier

**Affiliations:** 1 South Carolina Department of Natural Resources, Columbia, SC, USA

This research note presents observations on the temporal and spatial distribution of spiny dogfish, *Squalus acanthias*, in the western North Atlantic Ocean. Specifically the note documents presence and environmental data associated with the capture of spiny dogfish in the Back and Core Sound, North Carolina. While spiny dogfish have been documented later in the year (as late as June 1), associated environmental data have not been published. The findings published are of importance as they document the occurrence of mature female spiny dogfish in water temperatures above previously published thermal thresholds. Given recent management actions, these data are important as spiny dogfish may occur in their southern range well outside of periods where they are sampled.

These data are clear and concise and appropriate for publication as a research note. The data are straightforward, and I recommend indexing the article, although the authors should consider the following minor revisions.

***Abstract:*** Report ranges and mean lengths for spiny dogfish captured. Length at maturity should state length at 50% maturity. Significant digits for depth should be reported to the nearest tenth of a meter, this should also be corrected throughout the manuscript.

***Methods*:** Paragraph two, the authors should clarify if total length measurements are natural total length, or stretch total length. If they are estimating maturity based on length this could affect estimates.

***Results:*** Significant digits need to be corrected, again depths should reported to the nearest tenth of a meter, also DO significant digits are reported to the nearest tenth in one instance and the nearest one hundredth in the next.

For capture events it isn't necessary to report additional catch (batoids) as these data do not contribute to the note.

***Discussion:*** If temperature data are available for the Rulifson et al. (2012) reported June encounters of spiny dogfish, these data should be reported.

For Nammack *et al* reference, report what metric of length at maturity this is (median or length at 50% maturity)

I have read this submission. I believe that I have an appropriate level of expertise to confirm that it is of an acceptable scientific standard.
